# Structure of Coal-Derived Metal-Supported Few-Layer Graphene Composite Materials Synthesized Using a Microwave-Assisted Catalytic Graphitization Process

**DOI:** 10.3390/nano11071672

**Published:** 2021-06-25

**Authors:** Faridul Islam, Arash Tahmasebi, Rou Wang, Jianglong Yu

**Affiliations:** 1 Chemical Engineering, School of Engineering, The University of Newcastle, Callaghan, NSW 2308, Australia; faridul.islam@uon.edu.au (F.I.); arash.tahmasebi@newcastle.edu.au (A.T.); rou.wang@uon.edu.au (R.W.); 2 Suzhou Industrial Park Monash Research Institute of Science and Technology, Southeast University—Monash University Joint Graduate School, Suzhou 215000, China

**Keywords:** few-layer graphene, catalytic graphitization, microwave irradiation, iron oxide

## Abstract

Metal-supported few-layer graphene (FLG) was synthesized via microwave-assisted catalytic graphitization owing to the increasing demand for it and its wide applications. In this study, we quickly converted earth-abundant and low-cost bituminous coal to FLG over Fe catalysts at a temperature of 1300 °C. X-ray diffraction, Raman spectroscopy, transmission electron microscopy, and N_2_ adsorption–desorption experiments were performed to analyze the fabricated metal-supported FLG. The results indicated that the microwave-irradiation temperature at a set holding-time played a critical role in the synthesis of metal-supported FLG. The highest degree of graphitization and a well-developed pore structure were fabricated at 1300 °C using a S10% Fe catalyst for 20 min. High-resolution transmission electron microscopy analysis confirmed that the metal-supported FLG fabricated via microwave-assisted catalytic graphitization consisted of 3–6 layers of graphene nanosheets. In addition, the 2D band at 2700 cm^−1^ in the Raman spectrum of the fabricated metal-supported FLG samples were observed, which indicated the presence of few-layer graphene structure. Furthermore, a mechanism was proposed for the microwave-assisted catalytic graphitization of bituminous coal. Here, we developed a cost-effective and environmental friendly metal-supported FLG method using a coal-based carbonaceous material.

## 1. Introduction

Graphene consists of single layers of sp^2^–hybridized C atoms organized in a two-dimensional honeycomb matrix. Owing to its unique thermal, electronic, composite, and mechanical properties, graphene has recently attracted increasing attention as a critical material with numerous technological applications [1]. Moreover, numerous graphene synthesis methods using gaseous hydrocarbons as C sources have been developed, such as chemical vapor deposition (CVD) [2], chemical or electrochemical processes [3], epitaxial growth [4], and graphite-oxide exfoliation [5]. CVD is one of the most effective methods for synthesizing high-quality graphene [6]. However, solid C-like coal and food waste are increasingly used instead of gaseous hydrocarbons for graphene synthesis [7].

Utilization of coal as a fossil fuel has been linked to climate change [8], air pollution, and human health problems [9]; moreover, fossil fuel depletion is a global-scale phenomenon that impacts the environment [10]. These shortcomings have led to the development of an alternative efficient and clean graphene synthesis method [11]. Coal-based (anthracite, bituminous coal, and lignite) graphite was obtained at 3000 °C for 3 h with different microstructures such as honeycomb, monocrystalline, polycrystalline, spherical, and rod-like [12]. In addition, lignite-coal-derived synthetic graphite materials were prepared from 1200 to 2700 °C for 3 h [13]. Graphite is used as a raw material for graphene oxide (GO) production. It is synthesized by the modified Hummers method, which is related to time-consuming physicochemical reduction processes [5,14]. Moreover, flake graphite, which contains few impurities and consists of large crystals, is currently the primary source for GO production [15]. However, flake graphite poses a significant environmental threat owing to its natural scarcity and increasing demands for it in industrial and research sectors. Therefore, coaly graphite can be used as an inexpensive and abundant raw material for graphene production [16].

However, this process presents several disadvantages, as it is time and high-energy consuming and requires high temperatures; moreover, washing and drying are required to complete graphene production [17]. In addition, owing to their high heating efficiency and low energy consumption [18,19], microwaves are used as an alternative energy source in many fields, such as catalyst preparation [20] and environmental remediation [21]. Microwaves have many more benefits, such as heating uniformity [22], high production efficiency [23], rapid energy transfer [24], volumetric heating, a contactless process, and shorter device start-uptimes [25], which are used for several applications, such as nanomaterials synthesis (ZnO nanocomposites) [26], waste treatment (garbage and sludge) [27], within the analytical chemistry sector (ashing, extraction, and moisture analysis) [28,29], industrial processes (coal pre-treatment and processing, and polymeric composites) [30,31] and food processing (home cooking and pasteurization) [32,33]. Moreover, the nanopowder synthesis conducted through microwave devices is dependent on four different factors, such as the scale of the sample, pressure development, the sintering temperature, and other multi-functions. The most important factor is that the microwave technique provides a better reaction with controlling mode, and it has the ability to easily repeat results [34]. Microwave irradiation has been used to produce graphene materials from graphite oxide by converting microwave energy into heat [35]. The microwave-absorbing capacity of graphene is higher than that of GO; furthermore, unoxidized GO initiates a deoxygenation process, and the deoxygenated graphene acts as a microwave absorbent [36]. The low-temperature multilayer graphene growth was obtained from raw coal via catalytic (Cu foil) graphitization under microwave irradiation [37]. Kim et al. produced graphene via CO reduction using Al_2_S_3_. Furthermore, organic materials have been used to produce graphene dispersions [38,39]; however, the yield and concentration of graphene fabricated using organic materials are very low. As a result, fabricated graphene is expensive [40]. Moreover, graphite has been prepared by heating bituminous coal at a high temperature of 2800 °C [41], which was required to overcome the high graphitization enthalpy of graphite (~260 kcal mol^−1^) [42]. Therefore, researchers have used several catalysts, such as Fe, Ni, and Co, to decrease graphitization temperatures [43,44,45]. Kim et al. prepared C nanoparticles at 950 °C using Co as the promoter [46]. Moreover, porous graphitic carbon materials have been fabricated at low temperatures over Ni catalysts using activated carbon [44] and wood [47]. Additionally, iron gluconates, ferrocene, and Fe have been used to prepare several nanostructured graphitic materials [48,49]. Nevertheless, a cost-effective and time-efficient large-scale method for the industrial synthesis of few-layer graphene (FLG) with a well-controlled structure is still required. To overcome these drawbacks, in this study we developed a cost-effective, time-efficient catalytic method for the synthesis of metal-supported FLG from bituminous coal using a microwave temperature of 1300 °C. Furthermore, the fabricated metal-supported FLG was analyzed using powder X-ray diffraction (XRD), transmission electron microscopy (TEM), N_2_ adsorption–desorption, and Raman spectroscopy experiments.

## 2. Materials and Methods

### 2.1. Materials and Sample Preparation

Australian bituminous coal was used as a precursor for the preparation of FLG. Crushed raw coal was heated in an electric furnace (Carbolite-VST 12/300; Uk, max. temp. −1200 °C) at 600 °C (heating rate of 10 °C/min) under a continuous N_2_ flow for 30 min to remove volatile matter. The sample was crushed and sieved to <63 μm size fractions, which were subsequently steam-activated via carbonization at 800 °C (heating rate of 10 °C/min) for 30 min under a constant N_2_ flow of 250 mL/min. Then, the porous carbon samples were impregnated for 12 h with aqueous iron nitrate solutions (≥99.95% purity, Merck) of different Fe concentrations 2, 5, 10, and 20 (according to wt% of the porous sample), which served as catalysts. Next, a 0.1 M NH_4_OH solution was added to each solution and stirred for 30 min, followed by washing with deionized water. The mixtures were filtered and oven-dried at 110 °C. Next, the steam-activated Fe-loaded samples were divided into two sets and denoted as 800 °C–1300 °C (10% Fe loaded), and S2% Fe, S5% Fe, S10% Fe, and S20% Fe, respectively.

### 2.2. Microwave-Induced Graphitization

**Figure 1:**
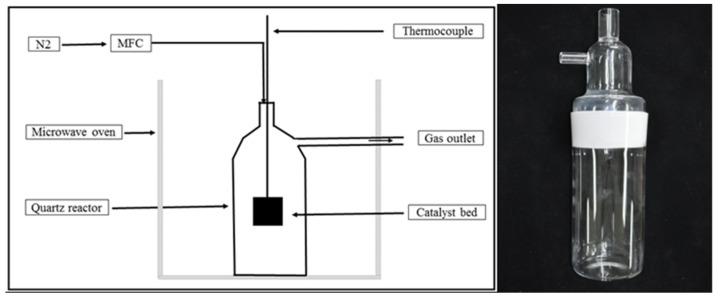
Schematic diagram of the catalytic graphitization setup (left); self-designed quartz reactor (right).

A self-designed quartz reactor (height of 360 mm, internal diameter of 80 mm, and thickness of 4 mm) with one inlet and one outlet (Figure 1) was placed inside a microwave oven (Tangshan Microwave Thermal Instrument CO. Ltd.; Beijing, China) with a microwave power output of 2000 W and a frequency of 2.45 GHz. In addition, a high-temperature type B thermocouple was inserted into the ceramic-crucible sample holder to measure sample temperatures at 2 s intervals. High-temperature insulation blankets surrounded the crucible to maintain a high internal temperature. For each experiment, 4 g of catalyst-loaded sample was used. N_2_ gas was flown (flow rate of 250 mL/min) through the reactor for 20 min before each experiment to create an inert reaction environment. The N_2_ flow rate (250 mL/min) and a microwave power output of 2000 W were fixed during experiments at target temperatures of 800–1300 °C.

Furthermore, the final temperature was maintained for 20 min to ensure sufficient time for reaction. The microwave-oven heating temperature, heating and cooling rate, and power system were fully controlled. The apparatus utilized online temperature monitoring to adjust the microwave power output to maintain the desired temperature. The catalyst-loaded graphitized samples were obtained from the microwave oven when the inside temperature was normal.

### 2.3. Characterization of Metal-Supported FLG Composite Materials

The morphology and structure of the metal-supported FLG samples were analyzed using a TEM 2100 (JEOL) TEM instrument at 200 kV. The porous properties of metal-supported FLG samples were examined by a N_2_ adsorption–desorption isotherm using a TriStar II 3020 (Micromeritics) at −196.15 °C. About 100 mg of samples were degassed at 400 °C for 4 h before starting the measurement. The Brunauer−Emmett−Teller method in a relative pressure range of 0.001–1 and the Barrett−Joyner−Halenda model were used to evaluate the specific surface area and pore-size distribution of the samples, respectively. A model (Bruker) XRD instrument with a Cu anode (λ = 0.154 nm) operated at a potential of 40 kV, and a current of 40 mA was used to determine the crystalline structure of samples in a scanning-angle range of 5−90°. Furthermore, the crystal size (La), stack height (Lc), and interlayer spacing (d_002_) of the samples were calculated using the Debye–Scherer equations and the g-factor values [50]. The structural order of the samples was evaluated using a Horiba tip-enhanced Raman spectroscopy–atomic force microscopy instrument with a wavelength of 532 nm, which was fitted with three bands (D ≈ 1350 cm^−1^, G ≈ 1580 cm^−1^, and 2D ≈ 2700 cm^−1^) to quantify the graphitization degree of the metal-supported FLG samples.

## 3. Results and Discussion

### 3.1. Morphology of Metal-Supported FLG Composite Materials

**Figure 2:**
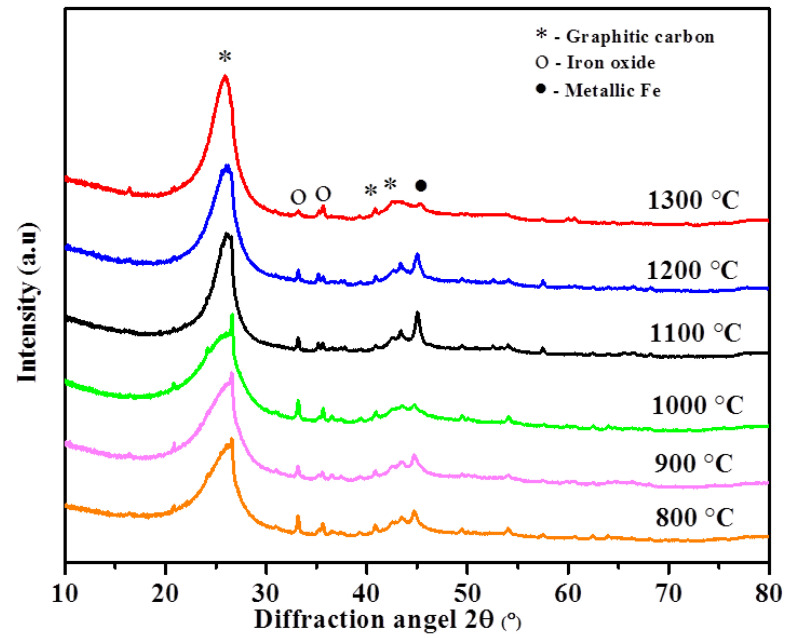
X-ray diffraction patterns of catalyst-loaded graphitized samples at different temperatures.

The XRD patterns of the catalytically graphitized composite materials using microwave radiation at different temperatures in the range of 800–1300 °C (10% Fe loaded) are presented in Figure 2. The sharp and intense peak at 2θ ≈ 26° in the XRD profile of the graphitized samples corresponded to the graphitic carbon. The peaks at 26° and 42.5° became sharper when the graphitization temperature was increased, due to aliphatic C chain breaking and the degradation of several functional groups [51]. According to Zhong et al., the strong diffraction peak in the graphite lattice represented the multilayer graphene accomplished with spherical particles [52]. The significant increase in diffraction-peak intensity with temperature increases in catalyst-loading bituminous coal specified the graphitic-layer ordering, supported by the most important factors, such as grain size, thickness, interlayer distance, graphitization degree (I_D_/I_G_), and g factor. The Scherrer and Bragg equations were used to calculate carbon-structure parameters. The La, Lc, interlayer spacing (d_002_), and g values are summarized in Table 1. Furthermore, the g value was measured using the Marie and Meiring equation. The sample graphitized at 1300 °C presented the largest crystal size and thickness of 3.28 and 7.06 nm, respectively, indicating that the catalyst promoted the growth of the staking height and graphite crystal particles. In addition, the interlayer spacing (d_002_) values of all samples were close to that of natural graphite (0.3354 nm), which is recommended for the growth of graphite structures [51].

The catalyst-loaded graphitized samples at 1300 °C represented an interlayer space (d_002_) and g-factor value of 0.3357 nm and 96.5%, respectively, which were higher than other temperatures.

**Figure 3:**
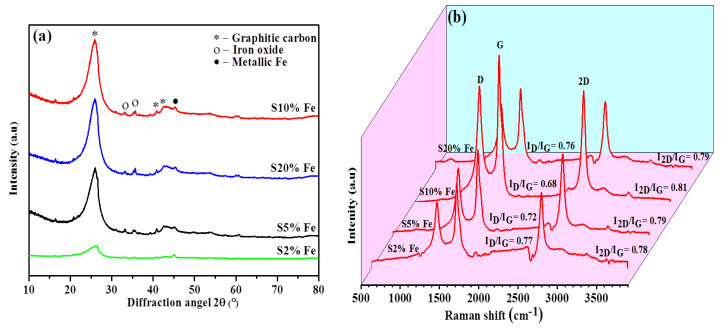
(**a**) X-ray diffraction patterns and (**b**) Raman spectra of different Fe-catalyst-loadings graphitized composite samples.

The most intense peak in the X-ray diffraction profile of the samples with different catalyst-loadings graphitized at 1300 °C was observed at 2θ ≈ 26° (Figure 3a). The intensity of the 26° peak in the XRD profile of the S10% Fe sample was higher than other Fe-catalyst loadings. The overall crystallinity of the samples catalytically graphitized at 1300 °C increased with increasing Fe-catalyst loading from S2% to S10%, and decreased when Fe-catalyst loading was further increased to S20%. This was ascribed to catalyst agglomeration and was confirmed by the calculated Lc, La, and d_002_ values presented in Table 2. The S10% Fe sample presented the lowest (I_D_/I_G_) value of 0.68, suggesting that its carbon structure was more ordered than those with other Fe loadings. Moreover, the grain size and thickness of the S20% Fe sample were smaller than those of the S10% Fe sample. The d_002_ value of the S10% Fe sample was 0.3357 nm, which was close to that of natural graphite (0.3354 nm), confirming that the S10% Fe sample presented a perfect crystal structure with a high g factor (96.5%). Moreover, the peak positions were directly linked with the d_002_ values of the carbon samples [53]. Typically, the d-spacing is correlated with the graphitization degree, which also depends on the graphitization time. Given the same processing time, catalytic microwave graphitization can lower the d-spacing value (0.336 nm) more effectively than thermal graphitization [54].

Furthermore, the metallic-Fe peak at 2θ ≈ 45.5° in the XRD profiles of the composite materials with different Fe-loadings fabricated via microwave-assisted catalytic graphitization (Figure 3a) indicated that iron oxide was reduced to metallic Fe during pyrolysis. In contrast, no stable ferric carbide (Fe_3_C) was formed, owing to the low amount of disordered carbon in the reaction mixture. Fe_3_C formed as an intermediate product of the conversion of iron oxide to metallic Fe during pyrolysis. The Fe nanoparticles acted as nuclei for the formation of a graphitic-layered structure. According to the Fe–C phase diagram, the eutectic point of Fe–C was 1148 °C [55]. The fast heating with an automated temperature-control mode facilitated the formation of metal-supported FLG under microwave radiation.

To confirm the FLG structure details, Raman spectroscopy is the most important technique for further supporting the obtained XRD results of fabricated samples. The D, G, and 2D Raman peaks at 1350, 1580, and 2700 cm^−1^, respectively, at an excitation wavelength of 532 nm were expressed as graphene and graphite positions (Figure 3b). Furthermore, the D peak was attributed to the disordered carbon structure and defects at the graphene surface, whereas the G and 2D peaks were attributed to the C=C bonds, a feature of graphene. Moreover, the sharp 2D peaks confirmed the metal-supported FLG structure of the analyzed samples [56]. Defects were present in all samples [57]. The I_D_/I_G_ ratio represented the degree of graphitization. A low (I_D_/I_G_) value indicates high graphitization. The I_D_/I_G_ value of the S10% Fe sample fabricated at 1300 °C (0.68) was the lowest among all analyzed samples (Table 2), suggesting that the S10% Fe sample presented a more ordered carbon structure than the samples with other Fe loadings. However, I_D_/I_G_ decreased at higher catalyst loadings as more disordered carbon structures formed, which was in agreement with the XRD results (Figure 3a). The 2D peak (2700 cm^−1^) was ascribed to the disordered structure of the samples and represented the number of graphene layers of each sample [56] (Table 2). The I_2D_/I_G_ value was correlated with the number of graphene layers. The S10% Fe sample presented the highest I_2D_/I_G_ value (0.81) among all analyzed samples. Moreover, the I_2D_/I_D_ ratio represented the overall crystallinity of the samples with a long carbon structure [58]. The I_2D_/I_D_ ratio gradually increased when the catalyst percentages increased (Table 2), indicating the long-range graphite structures. The highest I_2D_/I_D_ value (1.19) was achieved at 1300 °C for the S10% Fe sample, which was higher than those of the other analyzed samples (Table 2). However, the I_2D_/I_D_ value of the S20% Fe sample was lower than that of the S10% Fe sample, owing to catalyst agglomeration. In addition, TEM and high-resolution transmission electron microscopy (HRTEM) analyses were used to further confirm the morphology of the metal-supported FLG samples. Figure 3b also shows that a very broadly peaked background subtends all Raman bands, indicating a slight amorphization of the sample.

**Figure 4:**
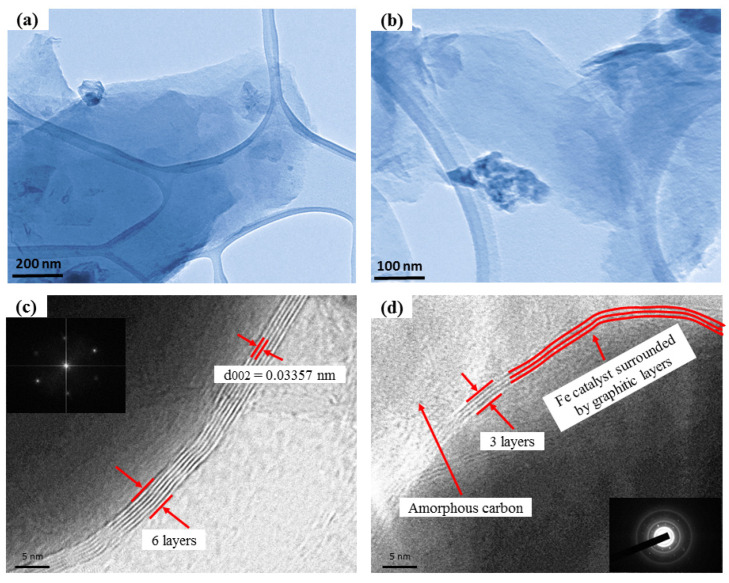
(**a**,**b**) TEM images of S10% Fe sample at different magnifications, (**c**,**d**) High-resolution transmission electron microscopy images of S10% Fe few-layer graphene sample. In addition, the inset (**c**) represents the FFT pattern with a hexagonal crystal structure, and the inset (**d**) shows the SAED configurations.

The TEM images of the metal-supported FLG samples are presented in Figure 4. Transition metals, such as Fe, Ni, and Cu, present outstanding catalytic graphitization properties due to their ionization potentials and d-electron structures [59]. These properties facilitate the dissolution of disordered carbon over catalysts at the supersaturation point and enable the growth of carbon layers into ordered structures via dissolution and precipitation [60]. A transparent few-layer graphene film over a larger area was clearly observed at different magnifications on a lacy carbon grid in Figure 4a,b, with the back-folding and wrinkles formed, as well as touching an edge due to the transfer process. H Okamoto studied that the graphene grew layer-by-layer, encompassing the Fe atoms at lateral directions [61]. This step followed the catalytic graphitization process that occurred via a dissolution–precipitation mechanism, as illustrated in the HRTEM image in Figure 4c,d. The HRTEM arbitrarily imaged edges of S10% Fe, revealing that the distorted nanosheets comprised about 3–6 layers of graphene nanosheets (Figure 4c,d). Furthermore, the HRTEM image confirmed interlayer spacing of around 0.34 nm (Figure 4c), which corresponded to the few-layered graphene nanosheet plane (002). The corresponding fast Fourier transform (FFT) (inset in Figure 4c) image represented the hexagonal spot pattern and demonstrated the sixfold symmetry of graphene features. The selected area electron diffraction (SAED) pattern (inset in Figure 4d) revealed the multiple sets of diffraction, which indicated the existence of few-layer graphene sheets [62,63].

**Figure 5:**
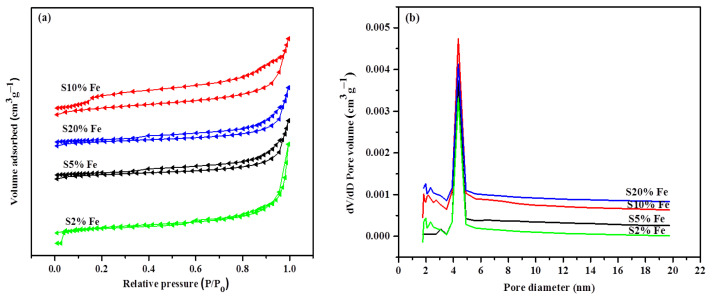
(**a**) N_2_ adsorption–desorption isotherms and (**b**) pore-size distribution of catalytically-graphitized few-layer graphene samples with different Fe loadings.

The textural properties of metal-supported FLG composites were determined using N_2_ adsorption–desorption isotherm analysis. The metal-supported FLG samples presented a type IV curve with a hysteresis loop in the relative pressure range of 0.001–1. This indicated the wide particle-size distribution range of the catalytically graphitized metal-supported FLG samples, as well as the presence of meso and micropores. The specific surface area of the S10% Fe sample (109.3 m^2^g^−1^) was the highest among all analyzed samples (Figure 5a) and was higher than that of natural graphite (5.5 m^2^g^−1^) [50], anthracite-based material (4.7–6.8 m^2^g^−1^) [64], and nickel-doped artificial graphite (4.65 m^2^g^−1^) [65]. In addition, the lowest surface area was that of S2% Fe, which was 51.57 m^2^g^−1^. The density functional theory calculations suggested that the pore size of FLG ranged between 1 and 20 nm; moreover, the mesopore distribution diagram featured a peak positioned at 4.7 nm, and the S10% Fe sample presented the largest particles among all analyzed samples (Figure 5b).

### 3.2. Proposed Mechanism of FLG Formation under Microwave Irradiation

**Figure 6:**
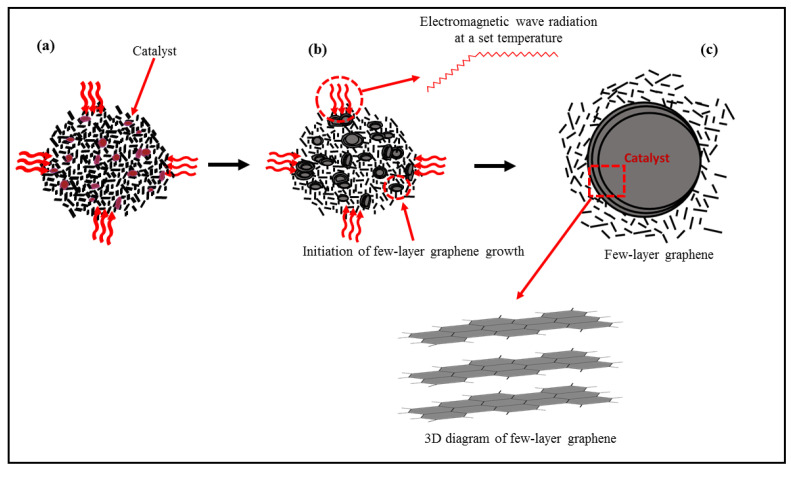
(**a**) Schematic diagram of catalyst particles in an amorphous carbon matrix under continuous microwave heating. (**b**) Initiation of few-layer graphene growth at a set temperature and time. (**c**) Microwave-induced graphitization and few-layer graphene growth around a catalyst particle.

Microwave-assisted catalytic graphitization is a complex process that is closely related to heating rate, catalyst absorption capacity, and holding time. It fabricated the supported FLG via the dissolution–precipitation mechanism. Therefore, it was hypothesized that microwave radiation played a significant role in the fabrication of metal-supported FLG. During microwave-assisted catalytic graphitization at a constant high heating rate and high temperature, the supersaturation point of the C matrix and Fe particles was reached quickly. Furthermore, the microwave enabled the layer-by-layer growth of graphene to be facilitated. The proposed FLG growth mechanism is presented in Figure 6. In this study, Fe(NO_3_)_3_·9H_2_O impregnated coal samples were pyrolyzed under an inert N_2_ atmosphere; the Fe(NO_3_)_3_·9H_2_O was decomposed to form iron oxide, and Fe particles were then formed via the carbothermal reduction in iron oxide. Next, amorphous carbon was precipitated on the oversaturated Fe particles to form graphite, a stable form of carbon, owing to the variation in adhesive forces between Fe particles and graphitic carbon, which played a dynamic role in the dissolution–precipitation mechanism [66]. In addition, the Bituminous coal was used as a source of C atoms and dissolved into the Fe droplets. When the concentrations of C and Fe droplets reached supersaturation, FLG grew layer-by-layer on the surface of Fe atoms, and amorphous C was converted into ordered C, owing to the change in Gibbs free energy [67,68]. A thick carbon layer was formed at a low heating rate because abundant C atoms were dissolved into the Fe-catalyst droplets. However, few-layer carbon was precipitated on the Fe-catalyst surface at a high heating rate, which favored the development of a carbon structure with a few-layer thickness [69]. Moreover, a microwave heating rate with a set temperature is a critical factor for the formation of metal-supported FLG. The schematic diagram of a catalyst loaded with an amorphous C structure is displayed in Figure 6a. At the eutectic point, amorphous carbon was dissolved into the Fe-catalyst particles under a constant microwave heating rate for 20 min (Figure 6b), initiating layer growth. Microwave-exposed radiation directly heated the particles with a controlled mode, which is a noble synchronization with the dissolution–precipitation of both catalyst and carbon. During heating, the active Fe-catalyst particles played a critical role and formed channels in the amorphous C matrix to make the graphitic layer [70]. A stable few-layer graphitic structure in a disordered carbon matrix and a three-dimensional diagram of metal-supported FLG is represented in Figure 6c. The aforementioned experimental results and proposed mechanism suggests that the number of graphene layers can be controlled by optimizing the temperature and catalyst-loading percentage. However, more studies should be performed to better understand the FLG growth mechanism.

## 4. Conclusions

In summary, metal-supported FLG materials were effectively fabricated from bituminous coal over Fe catalysts at 1300 °C using a microwave-assisted catalytic graphitization process. An innovative self-designed microwave-assisted test apparatus was used to conduct microwave graphitization tests. Catalyst loading and microwave temperature played significant roles in the fabrication of metal-supported FLG composite materials. The FLG growth process was induced when iron oxide was reduced to metallic Fe. Graphene nucleation and subsequent growth occurred via the dissolution of bituminous coal char within the Fe-catalyst droplets.

A unique FLG morphology with 3–6 layers of graphene nanosheets and a well-developed mesopore structure was achieved using Fe-catalyst loading of S10 wt%. The graphitization degree and pore size of the metal-supported FLG sample were 96.5% and 4.7 nm, respectively. Microwave graphitization required only 20 min and was much faster than the traditional heating method; moreover, the FLG precursor was abundant and inexpensive.

## Figures and Tables

**Table 1:** Structural properties of catalyst-loaded carbon materials fabricated via microwave-assisted catalytic graphitization at different temperatures.

Temperature (°C)/Parameter	800	900	1000	1100	1200	1300
Interlayer spacing (d_002_)(nm) ^1^	0.3366	0.3364	0.3368	0.3363	0.3362	0.3357
Crystallite size (Lc) (nm) ^1^	2.76	2.88	3.16	3.22	3.24	3.28
In-plane crystallite thickness (La) (nm)	5.95	6.20	6.82	6.95	6.98	7.06
g factor (%) ^2^	86.3	87.8	88.5	89.2	90.7	96.5

^1^ corresponding to the (002) graphitic plane in the X-ray diffraction profile of the sample. ^2^ graphitization degree calculated using the Marie and Meiring equation, as follows: g = (0.344 − d_002_)/(0.344 − 0.3354).

**Table 2:** Structural properties of composite materials with different Fe-loadings fabricated via microwave-assisted catalytic graphitization.

Fe Loading (%)/Parameter	S2	S5	S10	S20
Interlayer spacing (d_002_)(nm) ^1^	0.3367	0.3362	0.3357	0.3365
Crystallite size (Lc) (nm) ^1^	2.77	3.22	3.16	3.22
In-plane crystallite thickness (La) (nm)	5.59	6.94	7.06	6.68
I_D_/I_G_	0.77	0.72	0.68	0.76
I_2D_/I_G_	0.78	0.79	0.81	0.79
I_2D_/I_D_	1.01	1.10	1.19	1.03
Surface area (m^2^g^−1^)	51.57	63.31	109.3	85.12
g factor (%) ^2^	84.9	90.7	96.5	87.8

^1^ corresponding to the (002) graphitic plane in the X-ray diffraction profile of the sample. ^2^ graphitization degree calculated using the Marie and Meiring equation, as follows: g = (0.344 − d_002_)/(0.344 − 0.3354).

## Data Availability

Data is contained within this article.

## References

[1] Tan C., Cao X., Wu X.-J., He Q., Yang J., Zhang X., Chen J., Zhao W., Han S., Nam G.-H. (2017). Recent Advances in Ultrathin Two-Dimensional Nanomaterials. Chem. Rev..

[2] Kim K.S., Zhao Y., Jang H., Lee S.Y., Kim J.M., Kim K.S., Ahn J.H., Kim P., Choi J.Y., Hong B.H. (2009). Large-scale pattern growth of graphene films for stretchable transparent electrodes. Nature.

[3] Guo H.-L., Wang X.-F., Qian Q.-Y., Wang F.-B., Xia X.-H. (2009). A Green Approach to the Synthesis of Graphene Nanosheets. ACS Nano.

[4] Presser V., Heon M., Gogotsi Y. (2011). Carbide-Derived Carbons—From Porous Networks to Nanotubes and Graphene. Adv. Funct. Mater..

[5] Stankovich S., Dikin D.A., Piner R.D., Kohlhaas K.A., Kleinhammes A., Jia Y., Wu Y., Nguyen S., Ruoff R.S. (2007). Synthesis of graphene-based nanosheets via chemical reduction of exfoliated graphite oxide. Carbon.

[6] Cai W., Zhu Y., Li X., Piner R.D., Ruoff R.S. (2009). Large area few-layer graphene/graphite films as transparent thin conducting electrodes. Appl. Phys. Lett..

[7] Vijapur S.H., Wang D., Botte G.G. (2013). Raw Coal Derived Large Area and Transparent Graphene Films. ECS Solid State Lett..

[8] Letcher T.M. (2015). Climate Change: Observed Impacts on Planet EARTH.

[9] Brunekreef B., Holgate S.T. (2002). Air pollution and health. Lancet.

[10] Notarianni M., Liu J., Vernon K., Motta N. (2016). Synthesis and applications of carbon nanomaterials for energy generation and storage. Beilstein J. Nanotechnol..

[11] Wang G., Zhang L., Zhang J. (2012). A review of electrode materials for electrochemical supercapacitors. Chem. Soc. Rev..

[12] Qiu T., Yang J.-G., Bai X.-J. (2020). Preparation of coal-based graphite with different microstructures by adjusting the con-tent of ash and volatile matter in raw coal. Energy Sources Part A Recovery Util. Environ. Eff..

[13] Qiu T., Yang J.G., Bai X.J., Wang Y.L. (2019). The preparation of synthetic graphite materials with hierarchical pores from lignite by one-step impregna-tion and their characterization as dye absorbents. RSC Adv..

[14] Bi H., Wan S., Cao X., Wu X., Zhou Y., Yin K., Su S., Ma Q., Sindoro M., Zhu J. (2019). A general and facile method for preparation of large-scale reduced graphene oxide films with controlled structures. Carbon.

[15] Xian H., Peng T., Sun H., Wang J. (2015). Preparation of graphene nanosheets from microcrystalline graphite by low-temperature exfoliated meth-od and their supercapacitive behavior. J. Mater. Sci..

[16] Xian H., Peng T., Sun H., Wang J. (2015). Preparation, characterization and supercapacitive performance of graphene nanosheets from microcrys-talline graphite. J. Mater. Sci. Mater. Electron..

[17] Chen J., Li Y., Huang L., Li C., Shi G. (2015). High-yield preparation of graphene oxide from small graphite flakes via an improved Hummers method with a simple purification process. Carbon.

[18] Foo K.Y., Hameed B.H. (2011). Preparation of oil palm (*Elaeis*) empty fruit bunch activated carbon by microwave-assisted KOH activation for the adsorption of methylene blue. Desalination.

[19] Liu Q.S., Zheng T., Li N., Wang P., Abulikemu G. (2010). Modification of bamboo-based activated carbon using microwave radiation and its effects on the ad-sorption of methylene blue. Appl. Surf. Sci..

[20] Tian Z.Q., Jiang S.P., Liang Y.M., Shen P.K. (2006). Synthesis and Characterization of Platinum Catalysts on Multiwalled Carbon Nanotubes by Intermittent Microwave Irradiation for Fuel Cell Applications. J. Phys. Chem. B.

[21] Liu X., Quan X., Bo L., Chen S., Zhao Y., Chang M. (2004). Temperature measurement of GAC and decomposition of PCP loaded on GAC and GAC-supported copper catalyst in microwave irradiation. Appl. Catal. A Gen..

[22] Leonelli C., Lojkowski W. (2007). Main development directions in the application of microwave irradiation to the synthesis of nanopowders. Chem. Today.

[23] Leonelli C., Mason T.J. (2010). Microwave and ultrasonic processing: Now a realistic option for industry. Chem. Eng. Process. Process. Intensif..

[24] Schanche J.-S. (2003). Microwave synthesis solutions from personal chemistry. Mol. Divers..

[25] Wojnarowicz J., Chudoba T., Lojkowski W. (2020). A Review of Microwave Synthesis of Zinc Oxide Nanomaterials: Re-actants, Process Parameters and Morphoslogies. Nanomaterials.

[26] Singh A. (2010). Microwave synthesis, optical, structural and magnetic characterization of ZnO/Mn doped ZnO nanoparticles. J. Optoelectron. Adv. Mater..

[27] Vasudev H., Singh G., Bansal A., Vardhan S., Thakur L. (2019). Microwave heating and its applications in surface engineering: A review. Mater. Res. Express.

[28] Gaba M., Dhingra N. (2011). Microwave chemistry: General features and applications. Ind. J. Pharm. Edu. Res..

[29] Giberson R.T. (2001). Vacuum-Assisted Microwave Processing of Animal Tissues for Electron Microscopy. Springer Protocols Handbooks.

[30] Mishra R.R., Sharma A.K. (2016). A review of research trends in microwave processing of metal-based materials and op-portunities in microwave metal casting. Crit. Rev. Solid State Mater. Sci..

[31] Leonelli C., Veronesi P., Denti L., Gatto A., Iuliano L. (2008). Microwave assisted sintering of green metal parts. J. Mater. Process. Technol..

[32] Chandrasekaran S., Ramanathan S., Basak T. (2013). Microwave food processing—A review. Food Res. Int..

[33] Feng H., Yin Y., Tang J. (2012). Microwave Drying of Food and Agricultural Materials: Basics and Heat and Mass Transfer Modeling. Food Eng. Rev..

[34] Dąbrowska S., Chudoba T., Wojnarowicz J., Łojkowski W. (2018). Current trends in the development of microwave reactors for the synthesis of nanomaterials in la-boratories and industries: A review. Crystals.

[35] Zhu Y., Murali S., Stoller M.D., Velamakanni A., Piner R.D., Ruoff R.S. (2010). Microwave assisted exfoliation and reduction of graphite oxide for ultracapacitors. Carbon.

[36] Hu H., Zhao Z., Zhou Q., Gogotsi Y., Qiu J. (2012). The role of microwave absorption on formation of graphene from graphite oxide. Carbon.

[37] Masi C.A., Schumacher T.A., Hilman J., Dulal R., Rimal G., Xu B., Leonard B., Tang J., Fan M., Chien T. (2021). Converting raw coal powder into polycrystalline nano-graphite by metal-assisted microwave treat-ment. Nano Struct. Nano Objects.

[38] Hu L., Cheng Q., Chen D., Ma M., Wu K. (2015). Liquid-phase exfoliated graphene as highly-sensitive sensor for simultaneous determination of endocrine disruptors: Diethylstilbestrol and estradiol. J. Hazard. Mater..

[39] Wei P., Shen J., Wu K., Hu C. (2018). Tuning electrochemical behaviors of N-methyl-2-pyrrolidone liquid exfoliated graphene nanosheets by centrifugal speed-based grading. Carbon.

[40] Hernandez Y., Nicolosi V., Lotya M., Blighe F.M., Sun Z., De S., McGovern I.T., Holland B., Byrne M., Gun’Ko Y.K. (2008). High-yield production of graphene by liquid-phase exfoliation of graphite. Nat. Nanotechnol..

[41] Xing B., Zhang C., Cao Y., Huang G., Liu Q., Zhang C., Chen Z., Yi G., Chen L., Yu J. (2018). Preparation of synthetic graphite from bituminous coal as anode materials for high performance lithi-um-ion batteries. Fuel Process. Technol..

[42] Bonijoly M., Oberlin M. (1982). A possible mechanism for natural graphite formation. Int. J. Coal Geol..

[43] Lian W., Song H., Chen X., Li L., Huo J., Zhao M., Wang G. (2008). The transformation of acetylene black into onion-like hollow carbon nanoparticles at 1000 °C using an iron catalyst. Carbon.

[44] Liu Y., Liu Q., Gu J., Kang D., Zhou F., Zhang W., Wu Y., Zhang D. (2013). Highly porous graphitic materials prepared by catalytic graphitization. Carbon.

[45] Rodríguez-Manzo J.A., Pham-Huu C., Banhart F. (2011). Graphene growth by a metal-catalyzed solid-state transfor-mation of amorphous carbon. ACS Nano.

[46] Kim J., Lee J., Choi Y., Jo C. (2014). Synthesis of hierarchical linearly assembled graphitic carbon nanoparticles via catalytic graphitization in SBA. Carbon.

[47] Gutiérrez-Pardo A., Ramírez-Rico J., De Arellano-López A.R., Martínez-Fernández J. (2014). Characterization of porous graphitic monoliths from pyrolyzed wood. J. Mater. Sci..

[48] Sevilla M., Martinez-de Lecea C.S., Valdés-Solís T., Morallón E., Fuertes A.B. (2008). Solid-phase synthesis of graphitic carbon nanostructures from iron and cobalt gluconates and their uti-lization as electrocatalyst supports. Phys. Chem. Chem. Phys..

[49] Li S., Li F., Wang J., Tian L., Zhang H., Zhang S. (2018). Preparation of Hierarchically Porous Graphitic Carbon Spheres and Their Applications in Supercapacitors and Dye Adsorption. Nanomaterials.

[50] Yeh T.-S., Wu Y.-S., Lee Y.-H. (2011). Graphitization of unburned carbon from oil-fired fly ash applied for anode materials of high power lithium ion batteries. Mater. Chem. Phys..

[51] Wang T., Wang Y., Cheng G., Ma C., Liu X., Wang J., Qiao W., Ling L. (2020). Catalytic Graphitization of Anthracite as an Anode for Lithium-Ion Batteries. Energy Fuels.

[52] Zhong M., Yan J., Wu H., Shen W., Zhang J., Yu C., Li L., Hao Q., Gao F., Tian Y. (2020). Multilayer graphene spheres generated from anthracite and semi-coke as anode materials for lithi-um-ion batteries. Fuel Process. Technol..

[53] Kinoshita K. (1992). Electrochemical Oxygen Technology.

[54] Kim T., Lee J. (2016). Full graphitization of amorphous carbon by microwave heating. RSC Adv..

[55] Bystrzejewski M. (2011). Synthesis of carbon-encapsulated iron nanoparticles via solid state reduction of iron oxide nanoparti-cles. J. Solid State Chem..

[56] Malard L.M., Pimenta M.A., Dresselhaus G., Dresselhaus M.S. (2009). Raman spectroscopy in graphene. Phys. Rep..

[57] Pavoski G., Maraschin T., Fim F.D.C., Balzaretti N.M., Galland G.B., Moura C., Basso N. (2016). Few Layer Reduced Graphene Oxide: Evaluation of the Best Experimental Conditions for Easy Production. Mater. Res..

[58] Santangelo S., Messina G., Faggio G., Lanza M., Milone C. (2010). Evaluation of crystalline perfection degree of multi-walled carbon nanotubes: Correlations between thermal kinetic analysis and micro-Raman spectroscopy. J. Raman Spectrosc..

[59] Ōya A., Ōtani S. (1979). Catalytic graphitization of carbons by various metals. Carbon.

[60] Derbyshire F., Presland A., Trimm D. (1975). Graphite formation by the dissolution—Precipitation of carbon in cobalt, nickel and iron. Carbon.

[61] Okamoto H. (1992). The C-Fe (carbon-iron) system. J. Phase Equilibria Diffus..

[62] Zhang Y., Zhang L., Kim P., Ge M., Li Z., Zhou C. (2012). Vapor Trapping Growth of Single-Crystalline Graphene Flowers: Synthesis, Morphology, and Electronic Properties. Nano Lett..

[63] Liu L.H., Lyu J., Zhao T.K., Li T.H. (2015). Large area preparation of multilayered graphene films by chemical vapour deposition with high elec-trocatalytic activity toward hydrogen peroxide. Mater. Technol..

[64] Cameán I., LaVela P., Tirado J.L., Garcia A. (2010). On the electrochemical performance of anthracite-based graphite materials as anodes in lithium-ion batteries. Fuel.

[65] Ma C., Zhao Y., Li J., Song Y., Shi J., Guo Q., Liu L. (2013). Synthesis and electrochemical properties of artificial graphite as an anode for high-performance lithi-um-ion batteries. Carbon.

[66] Charon E., Rouzaud J.-N., Aléon J. (2014). Graphitization at low temperatures (600–1200 °C) in the presence of iron impli-cations in planetology. Carbon.

[67] Fitzer E., Kegel B. (1968). Reactions of Carbon Saturated Vanadium Carbide Melts with Different Order Carbons (*Catalytic Graphitization*). Carbon.

[68] Gür T.M. (2018). Review of electrical energy storage technologies, materials and systems: Challenges and prospects for large-scale grid storage. Energy Environ. Sci..

[69] Juang Z.-Y., Wu C.-Y., Lo C.-W., Chen W.-Y., Huang C.-F., Hwang J.-C., Chen F.-R., Leou K.-C., Tsai C.-H. (2009). Synthesis of graphene on silicon carbide substrates at low temperature. Carbon.

[70] Kiciński W., Norek M., Bystrzejewski M. (2013). Monolithic porous graphitic carbons obtained through catalytic graphiti-zation of carbon xerogels. J. Phys. Chem. Solids.

